# Movement disorders in cell surface antibody mediated autoimmune encephalitis: a meta-analysis

**DOI:** 10.3389/fneur.2023.1225523

**Published:** 2023-07-21

**Authors:** Pakeeran Siriratnam, Laura McArthur, Zhibin Chen, Peter Kempster, Mastura Monif

**Affiliations:** ^1^Neurosciences, The Central Clinical School, Monash University, Melbourne, VIC, Australia; ^2^Neurology, Alfred Health, Melbourne, VIC, Australia; ^3^Department of Neurology, The Royal Melbourne Hospital, Parkville, VIC, Australia; ^4^School of Public Health and Preventive Medicine, Monash University, Melbourne, VIC, Australia; ^5^Neurosciences Department, Monash Medical Centre, Clayton, VIC, Australia; ^6^School of Clinical Sciences of Medicine, Monash University, Clayton, VIC, Australia

**Keywords:** autoimmune encephalitis, seropositive, movement disorder, surface receptor, frequency

## Abstract

**Background:**

Autoimmune encephalitis (AE) is an increasingly recognized neuroinflammatory disease entity in which early detection and treatment leads to the best clinical outcomes. Movement disorders occur in AE but their characteristics are not well defined.

**Objectives:**

To identify the frequency, classification, and prognostic significance of movement disorders in AE.

**Methods:**

We conducted a systematic review and random-effects meta-analysis of movement disorders in cell surface antibody mediated AE. The frequency of any movement disorder as well as the classification of movement disorders in AE serotypes was determined. We looked at adults 18 years and older and included publications that described at least 10 cases. We used the following four electronic databases: Medline (Ovid), EMBASE (Ovid), APA Psychinfo, and Cochrane library.

**Results:**

A total of 1,192 titles and abstracts were reviewed. Thirty-seven studies were included in the final meta-analysis. At least one kind of movement disorder was present in 40% of the entire AE cohort, 53% with anti-NMDA receptor antibodies, 33% with anti-CASPR2 antibodies, 30% with anti-LGI1 antibodies and 13% with anti-GABA receptor antibodies. Dyskinesia was the commonest movement disorder in anti-NMDA antibody mediated AE and faciobrachial dystonic seizures were most frequent in anti-LGI1 antibody mediated AE. Patients with a movement disorder tended to have a higher mortality. The risk of bias in the included studies was mostly moderate or high.

**Conclusion:**

Movement disorders are common in AE and their identification, in conjunction with other clinical and paraclinical features, may facilitate earlier diagnosis. The prognostic implications of movement disorders in AE warrant further dedicated study.

**Systematic review registration:**

https://www.crd.york.ac.uk/prospero/, identifier: CRD42023386920.

## 1. Introduction

Some movement disorders for which an immunological basis is now recognized have a long history. Sydenham wrote about his eponymous choreiform disorder in the 17th century, though links with pharyngitis and rheumatic fever were not made until almost 200 years later (1). Encephalitis lethargica was a common cause of parkinsonism and other abnormal movements in the first half of the 20th century. The entity itself, and its relationships with the roughly contemporaneous influenza pandemic, remain enigmatic (2). Cerebellar deficits were established as remote effects of cancer through clinico-pathological research (3). A modern era of immune-mediated movement disorders commenced when associations with neuroglial humoral autoimmunity were first described (4, 5).

Medical knowledge crystallizes faster around a laboratory disease identifier than by classification of clinical phenomenology alone. These anti-neuronal antibodies have led to new frameworks of nomenclature, and to insights into the immunological and molecular pathologies of autoimmune movement disorders. The area is complex, with regular publications on new antibodies and new clinical associations. Specific movement disorders can be prominent features of autoimmune encephalitis (AE), and authoritative review articles highlight their use as diagnostic clues (6). More frequently, however, movement abnormalities are present as part of a wider autoimmune encephalopathy. Movement disturbances themselves can be admixed, diluting the ability to discern ‘typical' serotype-phenotype correlations (7).

AE is uncommon but not rare. With an estimated prevalence of 13.7 per 100,000, it appears to be at least as prevalent as infectious encephalitis, particularly in younger age groups (8). There are several types of pathogenic antibodies in AE—antibodies to cell surface proteins; antibodies to intracellular synaptic proteins; antibodies that target other intracellular antigens in combination with T-cells; and antibodies associated with non-neurological autoimmune disorders (9). Identifiable antibodies that target proteins expressed on nerve cell surfaces—anti-N-methyl-d-aspartate receptor (NMDAR), anti-leucine-rich glioma-inactivated 1 protein (LGI1), anti-contactin-associated protein-like receptor 2 (CASPR2), anti-gamma aminobutyric acid receptor (GABAR)—define an important, relatively treatment-sensitive subgroup of seropositive AE (8). Antibodies against intracellular epitopes, on the other hand, are more common in paraneoplastic AE. There is restricted serotype-specific information from large datasets about movement disorders in AE. More precise knowledge could reduce delays in clinical recognition and diagnosis, which have major implications for patient outcomes (10).

The purpose of this meta-analysis is to clarify the frequency, classification, clinical associations and prognostic significance of movement disorders in adult AE. Its focus will be on cell surface antibody mediated AE, where the evidence for antibody pathogenicity is strongest and immunotherapy tends to achieve its best results (8). To the best of our knowledge, this is the first meta-analysis focusing on movement disorders in AE.

## 2. Methods and materials

This study follows the Preferred Reporting Items for Systematic Reviews and Meta-Analyses (PRISMA) guidelines (11). It is registered with PROSPERO 2022 CRD42023386920.

### 2.1. Eligibility criteria

#### 2.1.1. Included studies

We selected original research published prior to 10 August 2022 that reported on at least 10 patients aged 18 years or older with a diagnosis of AE supported by detection of pathologically significant autoantibodies. Studies had to describe clinical features of the illness. Ten was chosen as the minimum number of patients in a publication for meaningful statistics, an approach used in other AE review articles (12). All study designs (i.e., retrospective, prospective, case series and cohorts) were included. We did not limit eligibility by country, gender or upper age limit.

#### 2.1.2. Excluded studies

Pediatric AE differs from the adult disorder in many respects, so we excluded studies that reported only on children. For publications on mixed populations of adults and children, we included information pertaining to adults if the ≥18 years minimum number criterion was met. Some papers reported on mixed age groups without listing precise numbers of adults and children. We did not exclude a paper if we judged that adults were likely to have made up 10 or more patients.

Studies where only abstracts were available and non-English studies published without translation were excluded. Some papers reported on the same study cohort. In these instances, we chose the paper with the most comprehensive and recent data, and ignored others. We excluded studies of two conditions associated with cell surface antibodies—stiff person syndrome, not usually considered a type of AE; and Morvan's syndrome, essentially a peripheral neuromuscular disorder. We also excluded studies that concentrated on antibodies directed against intracellular proteins and the paraneoplastic disorders commonly associated with them. These have different clinical and prognostic profiles to cell surface antibody-related AE (13).

### 2.2. Literature search

Literature searches were conducted using the following electronic databases: Medline (Ovid) from 1946 to August 2022, EMBASE (Ovid) from 1974 to August 2022, and APA Psychinfo from 1987 to August 2022. We also hand searched the Cochrane library, though this did not yield additional articles. Combinations and synonyms of various movement disorders as well as the different cell surface antibodies were used as search terms. The search strategy is detailed in Appendix 1, including an exhaustive list of search terms.

### 2.3. Study selection and risk of bias

Two reviewers (PS and LM) independently performed title/abstract and full text screening to determine eligible papers; disagreements were adjudicated by the two senior authors (MM and PK). Risk of bias and study quality were assessed using the Newcastle-Ottawa scale for case control and cohort studies (14), an adapted version of the Newcastle-Ottawa Scale for cross-sectional studies (15), and the quality assessment tool developed by Monga et al. for case series (16). We used our previously published matrix (12) to classify each study as good, fair or poor quality (Supplementary Table 1).

### 2.4. Data extraction

Data were extracted independently by the two reviewers for studies that met the inclusion and exclusion criteria; discrepancies were adjudicated by the two senior authors. Each eligible study underwent a detailed review. Clinical information, including laboratory results, were collated. To be subject to meta-analysis, a clinical characteristic had to be represented in at least 3 articles.

#### 2.4.1. Classification of movement disorders

While all selected studies contained information on movement disorders, the use of descriptive terms varied considerably. None of the papers provided extensive details on classification, and most did not provide data on individuals.

We used the following terms to encompass the various descriptors of movement disorders:

- Chorea: for chorea, choreiform, choreoathetosis.
- Ataxia: for ataxia, cerebellar.
- Parkinsonism, dystonia and myoclonus, where they appeared, were used consistently. Only parkinsonism exceeded the threshold for statistical analysis.
- Faciobrachial dystonic seizures (FBDS) was consistent terminology for this uncommon movement disorder.
- Dyskinesia is not a tightly defined term, which is context dependent. In, for instance, Parkinson's disease, it is an umbrella description of involuntary movement that may comprise elements of chorea, dystonia and even myoclonus. Under ‘dyskinesia' we included use of that term as well as generic mention of involuntary movements that implied a hyperkinetic movement disorder. Orofacial dyskinesia was included here.

As several papers did not specify the type of movement disorder, we estimated the minimum and maximum possible numbers of patients who had any movement disorder in all studies. This attempts to address movement disorder terminologies that may not have been mutually exclusive and individuals with multiple movement disorders that were not accurately reported. ‘Any movement disorder (minimum)' in a study was derived using the number of the largest movement disorder subtype; ‘any movement disorder (maximum)' was the summation of all the numbers of movement disorder subtypes or the total study sample size, whichever was smaller. Given the rarity of the GABAR mediated subtype, AE attributed to either GABA-A or GABA-B receptor antibodies were combined in one category.

#### 2.4.2. Other clinical features

We collected information about prodromal symptoms, cognitive effects, sleep disturbances, sensory symptoms, autonomic symptoms, abnormal conscious state, seizures, psychiatric features, speech and associated neoplasms. Basic demographic details (sex, age and country of residence) were also recorded.

#### 2.4.3. Timing of clinical features

In many publications, it was not clear whether certain clinical features were present before or after treatment. When two time points of information were provided, we included the subset with the highest frequency as the better guide to the occurrence of movement disorders.

#### 2.4.4. Ancillary investigations

Where available, we collected data on cerebrospinal fluid (CSF) microscopy and biochemistry, magnetic resonance imaging of brain (MRI B) and electroencephalogram (EEG) results. In view of a large variability in descriptions of MRI B and EEG findings, we registered any reported departure from normal as abnormal. On the basis of limited and variable reporting of CSF analysis, we classified CSF microscopy as either normal or pleocytosis (more than 5 white cells/μL where cell counts were provided), and CSF protein as either normal or elevated (>0.45 mg/mL where numerical results were given, although some other articles defined elevated protein as >0.5 mg/mL).

#### 2.4.5. Treatments

We collected data on the first line (corticosteroids, plasma exchange and intravenous immunoglobulin) and subsequent therapies.

#### 2.4.6. Outcome measures

Where available, we collected data on deaths, relapses and modified Rankin Scale (mRS) assessments of neurological disability before and after treatment. Several papers reported outcomes as ‘good', ‘favorable' or ‘excellent'; others gave the mean mRS or a change in mRS. To minimize the risk of reporting bias or selection bias in the interpretation of mRS, we only included mRS when an average score was provided pre- and post-treatment.

#### 2.4.7. AE and associated outcomes

Since we did not have individual patient data, we analyzed the relationship between the proportion of patients with movement disorders and the proportion of patients with various outcomes (mRS, relapse and mortality).

### 2.5. Statistical analysis

When at least 3 studies were available, we performed random effect meta-analyses with DerSimonian and Laird approach to estimate the proportions of movement disorders, other clinical features, investigation findings and outcomes in patients with AE and its subtypes (17). I^2^ statistic was used to quantify the magnitude of between-study heterogeneity. Random effects meta-regressions were used to explore whether AE subtype, age, sex, year of publication and national income level contributed to between-study heterogeneity in proportions of movement disorders. Heterogeneity that could be explained by a factor was quantified using R^2^ statistic. We also explored whether the presence of a movement disorder was associated with other clinical features. Funnel plot visual analysis and Egger's test were used on Freeman-Tukey double arcsine transformed data to evaluate small study effect and publication bias for movement disorders (18, 19). Statistical significance level was set at *p* < 0.05. Holm-Bonferroni (HB) method was used to correct for multiple comparisons where applicable (20). All statistical tests were conducted using Stata version 16.1 (StataCorp, College Station, Texas, USA) where meta-analysis of proportions was performed using user-written package ‘metaprop' (21).

#### 2.5.1. Data availability policy and statement

Data not provided in the article may be shared (anonymized) at the request of any qualified investigator for purposes of replicating procedures and results.

## 3. Results

### 3.1. Search results

**Figure 1:**
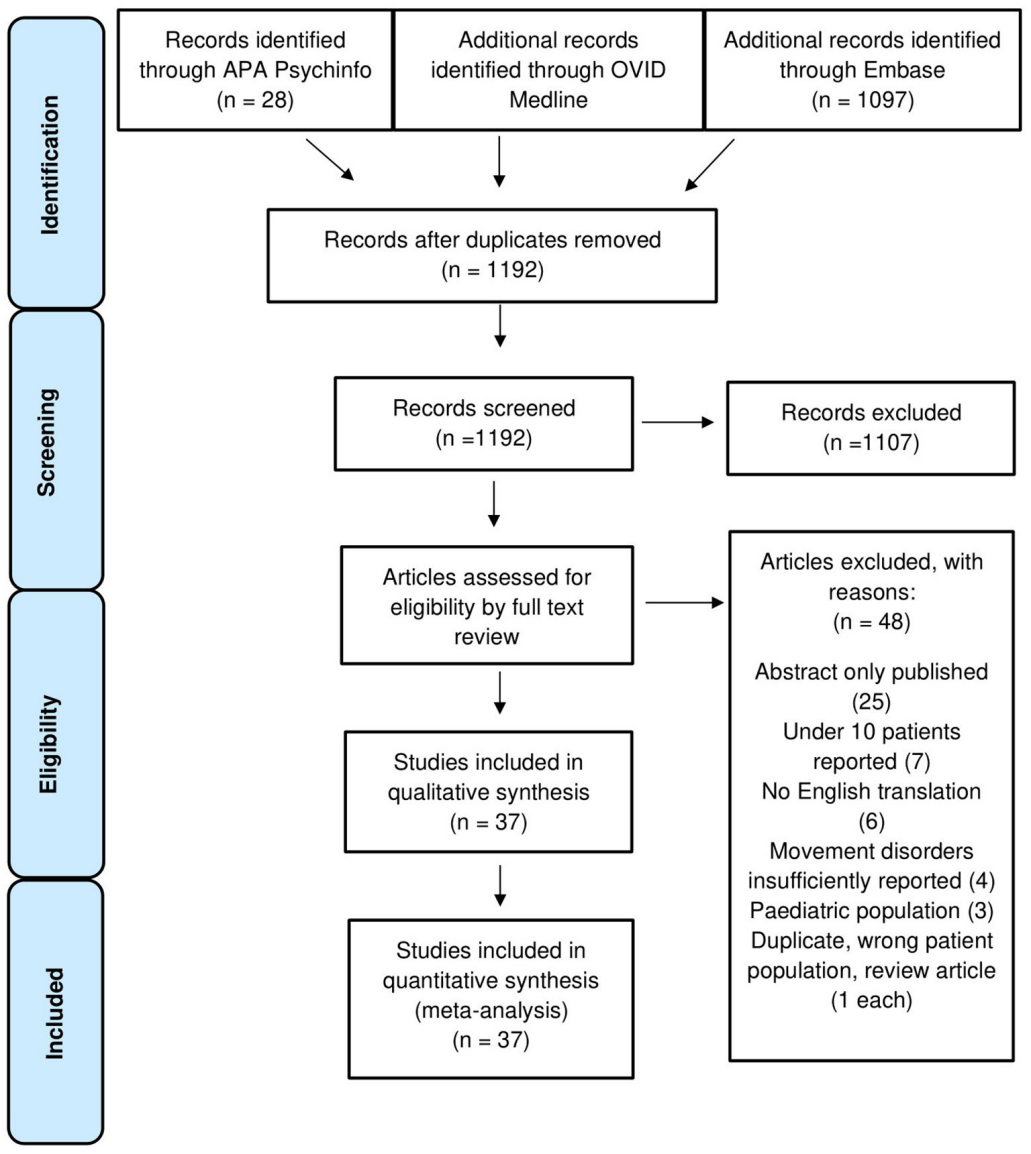
PRISMA flowchart demonstrating inclusion and exclusion criteria in the systematic review.

A total of 1,192 studies were inspected at abstract level, of which 1,107 were excluded. Eighty-five full texts were screened and a total of 37 studies were accepted for qualitative synthesis and meta-analysis. A flowchart of the study selection process is displayed in Figure 1 (22–58).

### 3.2. Qualitative synthesis

Of the 37 eligible studies, there were 22 case series, 13 cohort studies, 1 case control study and 1 cross-sectional study. All used one of, or a combination of: chart review, written questionnaire or interview. Data from 2,663 patients were included, with NMDAR mediated AE accounting for the majority (*n* = 1,343; 50%), followed by LGI1 (*n* = 729; 27%), CASPR2 (*n* = 257; 10%) and GABAR (*n* = 143; 5%). The remaining 191 patients (7%) had other, multiple, or unspecified AE subtypes. Stratified by AE autoantibody, the demographics of the 37 included publications are presented in Table 1. Detailed demographics from individual studies appear in Supplementary Table 2.

**Table 1:** Demographics of 2,663 patients from 37 studies, stratified by AE autoantibody.

**Autoantibody**	**Number of studies**	**Number of patients**	**Weighted average age (years)^∧^**	**Females (%)^#^**
Anti-NMDAR	22	1,343	26	720 (57)
Anti-LGI1	15	729	59	262 (37)
Anti-CASPR2	6	257	62	48 (19)
Anti-GABAR	6	143	56	57 (40)
Anti-IgLON5	2	85	62	38 (45)
Anti-GAD	3	5	35	1 (33)
Anti-VGKC	1	2	57	1 (50)
Both Anti-LGI1 and anti-CASPR2	3	21	52	8 (38)
Other/Unspecified^*^	5	78	49	35 (45)

NMDAR, anti-N-methyl-d-aspartate receptor; CASPR2, anti-contactin associated protein-like 2; GABAR, gamma-aminobutyric-acid; GAD, glutamic acid decarboxylase; IgLON5, immunoglobulin-like cell adhesion molecule 5; LGI1, anti-leucine-rich glioma-inactivated 1; VGKC, voltage-gated potassium channel.

# 89 patients with anti-NMDAR antibody mediated AE from two studies, 25 patients with anti-LGI1 antibody mediated AE from two studies, and two patients with anti-GAD antibody mediated AE from one study were missing sex information and excluded when calculating the proportions of female.

∧ Calculated from a combination of means and medians reported.

* Other/unspecified include publications which mentioned anti-anti-thyroid peroxidase and anti-zinc finger protein 4.

### 3.3. Quantitative synthesis (meta-analysis)

**Figure 2:**
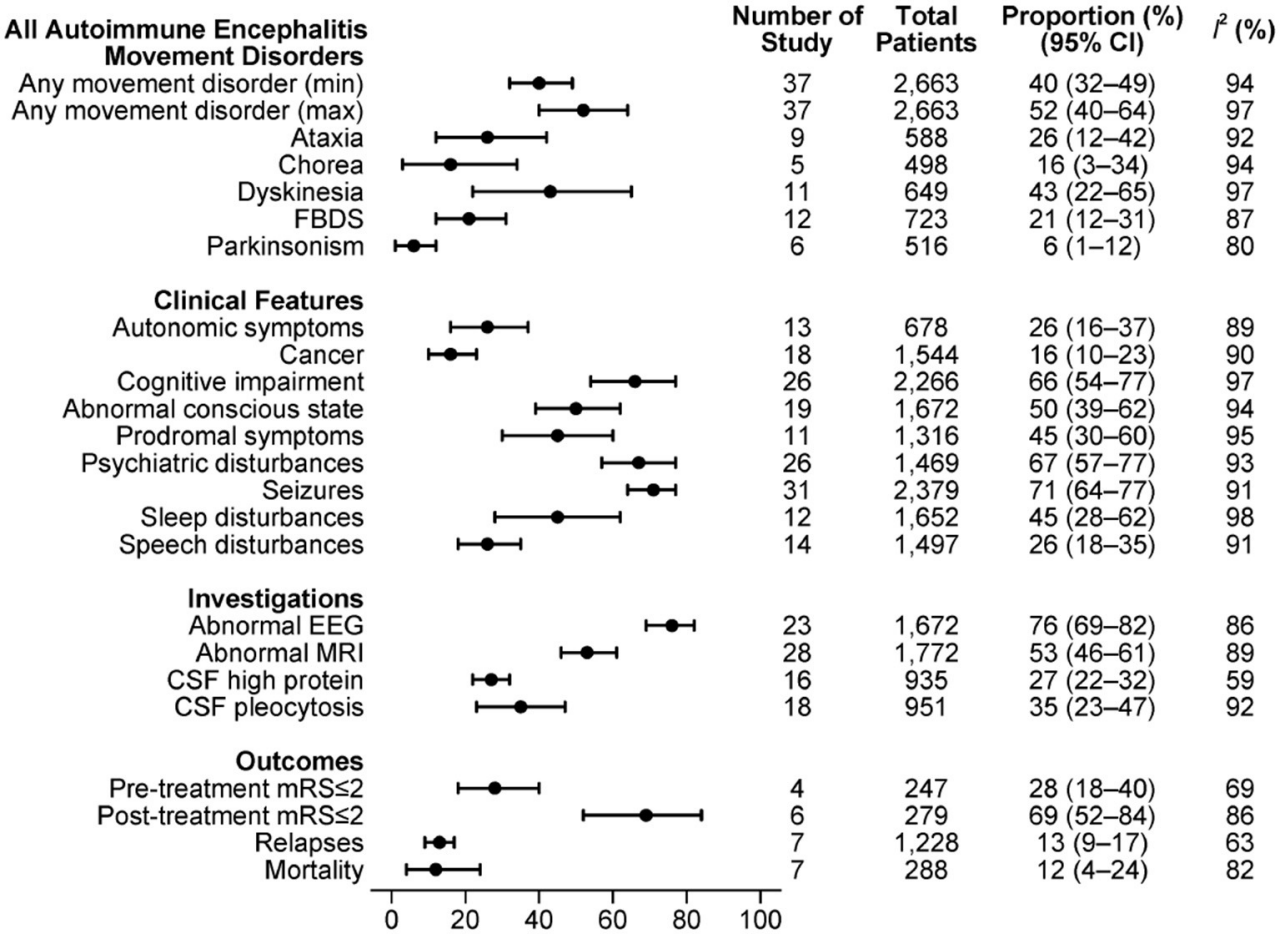
Clinical features in all included subtypes of AE.

The combined estimations of proportions of movement disorders with respect to other clinical features, investigation findings and outcome measures in the overall sample of AE are shown in Figure 2.

A minimum of 40% [95% confidence interval (CI): 32–49%, I^2^ = 94%] of patients experienced at least one type of movement disorder, with maximum estimation of 52% (95% CI: 40–64%, I^2^ = 97%). Among studies that reported movement disorder classifications, dyskinesia was the most frequent (43%, 95% CI: 22–65%, I^2^ = 97%). By individual paper, FBDS (12 studies) and dyskinesia (11 studies) were the most frequently documented movement disorders.

Clinical features such as seizures (31/37), psychiatric disturbance (26/37) and cognitive change (26/37) were described in most of these studies. Seventy-one percent of AE patients had epilepsy (95% CI: 64–77%, I^2^ = 91%), 67% had psychiatric features (95% CI: 57–77%, I^2^ = 93%) and 66% had cognitive impairment (95% CI: 54–77%, I^2^ = 97%).

MRI B (28/37) and EEG (23/37) results were reported in the majority of the studies, and about half reported CSF findings (19/37). We estimated that two-thirds of the AE patients had an abnormal EEG (76%, 95% CI: 69–82%, I^2^ = 86%), more than half had an abnormal MRI B (53%, 95% CI: 46–61%, I^2^ = 89%), and more than a third had either CSF pleocytosis or raised protein.

Many articles were case series that did not give outcome measures. Among studies that reported outcomes, more than two-thirds of patients had post-treatment mRS≤2 (69%, 95% CI: 52–84%, I^2^ = 86%), while only 28% had mRS≤2 before treatment (95% CI: 18–40%, I^2^ = 69%). The estimated relapses rate was 13% (95% CI: 9–17%, I^2^ = 63%), with an overall mortality of 12% (95% CI: 4–24%, I^2^ = 82%).

### 3.4. Meta-regression and heterogeneity

The minimum proportion of any movement disorder was positively associated with autonomic symptoms, cancer, abnormal conscious state, psychiatric disturbance, sleep disruption and speech deficit. Similar associations were found between the maximum proportion of any movement disorder and all of the above clinical features except cancer (Table 2).

**Table 2:** Associations between any movement disorder and clinical features.

		**Autonomic symptoms**	**Cancer**	**Cognitive impairment**	**Abnormal conscious state**	**Prodromal symptoms**	**Psychiatric disturbance**	**Seizures**	**Sleep disturbance**	**Speech disturbance**
Any movement disorder (min)	Coefficient	1.25	0.68	0.35	1.54	0.65	1.32	0.59	1.81	1.64
	(95% CI)	(0.45–2.05)	(0.05–1.30)	(−0.84–1.55)	(0.98–2.10)	(−0.42–1.71)	(0.57–2.08)	(−0.02–1.21)	(0.64–2.98)	(1.24–2.04)
	*p*-value	0.002	0.033	0.56	< 0.001	0.23	< 0.001	0.057	0.002	< 0.001
Any movement disorder (max)	Coefficient	1.02	0.54	0.48	1.30	0.65	0.83	0.44	1.10	1.21
	(95% CI)	(0.36–1.68)	(−0.06–1.13)	(−0.29–1.25)	(0.81–1.79)	(−0.42–1.71)	(0.12–1.54)	(−0.10–0.99)	(0.10–2.09)	(0.77–1.66)
	*p*-value	0.003	0.078	0.22	< 0.001	0.23	0.021	0.11	0.03	< 0.001

CI, confidence interval.

Considerable between-study heterogeneity (I^2^≥75%) was found for almost all features, exceptions being proportions of elevated CSF protein (I^2^ = 59%), relapses (I^2^ = 63%) and pre-treatment mRS≤2 (I^2^ = 69%). Compared with studies that only included LGI1 mediated AE, studies that only included the NMDAR type had a lower proportion of FBDS (coefficient = −1.02, HB-corrected *p* = 0.006) and a higher proportion of dyskinesias (coefficient = 1.40, HB-corrected *p* = 0.002). The AE subtype variable could explain majority of between-study heterogeneities for these two movement disorders (R^2^ = 54% and R^2^ = 72%, respectively). No factors were found to be associated with the proportions of ataxia and parkinsonism in AE nor attributed to their between-study heterogeneities (Supplementary Table 3).

### 3.5. AE and associated outcomes

The proportion of any movement disorder was positively associated with post-treatment mRS score under 2 (coefficient = 1.53 and *p* = 0.013 for minimum occurrence of movement disorders; coefficient = 1.81 and *p* < 0.001 for maximum movement disorders). The data for pre-treatment mRS is conflicting, as there is a positive association with minimum occurrence of any movement disorder (coefficient = 1.79 and *p* = 0.022) but a negative association for maximum movement disorder (coefficient = −0.74 and *p* = 0.6). There was a positive association between mortality (coefficient = 1.61 and *p* = 0.004 and minimum movement disorder; coefficient = 1.26 and *p* < 0.001 for maximum movement disorder—both adjusted for age and sex). Mortality was negatively associated with relapses (coefficient = −0.78, *p* < 0.001) for minimum and maximum (coefficient = −0.69, *p* < 0.001) movement disorders (Table 3).

**Table 3:** Associations between any movement disorder and outcome measures.

		**Pre-treatment mRS ≤ 2**	**Post-treatment mRS ≤ 2**	**Relapses**	**Mortality**	
					**Raw**	**Adjusted with age and sex**
Any movement disorder (min)	Coefficient	1.79	1.53	−0.78	1.23	1.61
	(95% CI)	(0.26–3.32)	(0.32–2.73)	(−1.22−0.33)	(−0.36–2.83)	(0.50–2.71)
	*p*-value	0.022	0.013	< 0.001	0.13	0.004
Any movement disorder (max)	Coefficient	−0.74	1.81	−0.69	1.18	1.26
	(95% CI)	(−3.47–1.99)	(1.20–2.41)	(−1.06–−0.31)	(0.16–2.21)	(0.69–1.83)
	*p*-value	0.60	< 0.001	< 0.001	0.024	< 0.001

CI, confidence interval.

### 3.6. Subgroup analysis

**Figure 3:**
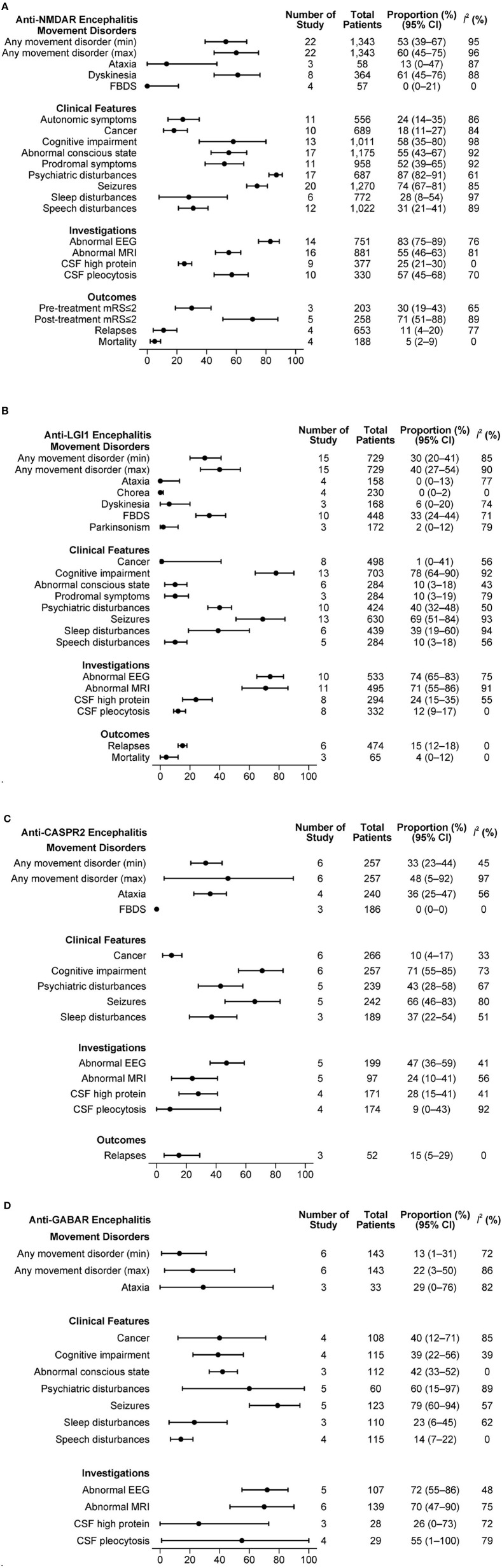
**(A)** Clinical features in anti-NMDAR antibody mediated AE. **(B)** Clinical features in anti-LGI1 antibody mediated AE. **(C)** Clinical features in anti-CASPR2 antibody mediated AE. **(D)** Clinical features in anti-GABAR antibody mediated AE.

The combined estimations of clinical features in antibody-specific subgroups of AE are presented in Figures 3A–D.

#### 3.6.1. NMDAR mediated AE

From 22 studies with a total of 1,343 patients, a minimum of 53% and a maximum of 60% of patients experienced at least one type of movement disorder. Dyskinesia was frequent (61%, 95% CI: 45–76%, I^2^ = 88%), while FBDS was rarely reported (0%, 95% CI: 0–21%, I^2^ = 0%). A substantial proportion of patients had psychiatric disturbances (87%, 95% CI: 82–91%, I^2^ = 61%), seizures (74%; 95% CI: 67–81%, I^2^ = 85%) and abnormal EEG (83%, 95% CI: 75–89%, I^2^ = 76%). The relapse rate reported in 4 studies that included 653 patients was 11% (95% CI: 4–20%, I^2^ = 77%). In another set of 4 studies with 188 patients, the mortality was 5% (95% CI: 2–9%, I^2^ = 0%).

#### 3.6.2. LGI1 mediated AE

Movement disorders were present in at least 30% and up to 40% of 729 patients from 15 studies that described LGI1 mediated AE. FBDS was the dominant movement disorder (33%, 95% CI: 24–44, I^2^ = 71%). Other movement disorders were rarely reported. Clinical features such as cognitive disturbances (78%, 95% CI: 64–90%, I^2^ = 92%) and seizures (69%; 95% CI: 51–84%, I^2^ = 93%) were commonly present. Cancer was rare (1%, 95% CI: 0–41%, I^2^ = 56%). Many patients had an abnormal EEG (74%, 95% CI: 65–83%, I^2^ = 75%) and/or abnormal MRI B (71%, 95% CI: 55–86%, I^2^ = 91%). In a set of 6 studies with 474 patients, the relapse rate was 15% (95% CI: 12–18%, I^2^ = 0%). The mortality reported in 3 studies with 66 patients was 4% (95% CI: 0–12%, I^2^ = 0%).

#### 3.6.3. CASPR2 mediated AE

Among 257 patients from 6 studies, any movement disorder was reported in 33 to 48%. Of the movement disorders reported in at least three studies, ataxia was present in 36% (95% CI: 25–47%, I^2^ = 56%). Cognitive disturbances (71%, 95% CI: 55–85%, I^2^ = 73%) and seizures (66%, 95% CI: 46–83%, I^2^ = 80%) were common. The relapse rate was 15% (95% CI: 5–29%, I^2^ = 0%) in 52 patients from 3 studies.

#### 3.6.4. GABAR mediated AE

An estimated 13 to 22% of the patients had at least one movement disorder in the 143 patients from 6 studies. Ataxia was the only movement disorder type registered by at least 3 studies, being present in 29% of the patients (95% CI: 0–76%, I^2^ = 82%). Seizures (79%; 95% CI: 60–94%, I^2^ = 57%) and psychiatric disturbances (60%; 95% CI: 15–97%, I^2^ = 89%) were the other common clinical features. Based on 108 patients from 4 studies, we estimated that 40% of the patients had cancer (95% CI: 12–71%, I^2^ = 85%). The proportions of patients with abnormal EEG or MRI B were high (≥70% for both). No outcome measures were reported in at least 3 studies.

### 3.7. Small study effect and publication bias

Small study effect was initially detected for parkinsonism (*p* = 0.002). Given the high between-study heterogeneity for parkinsonism (I^2^ = 82%), age, which could account for some heterogeneity (R^2^ = 38%), was included as a moderator in Egger's test. This attempts to assess small-study effects for reasons other than heterogeneity. No evident small study effect was then found (*p* = 0.22). No small study effect was found for other types of movement disorders aside from parkinsonism. The funnel plots for study effect for any movement disorder (minimum and maximum) are presented in Supplementary Figures 1A, B.

### 3.8. Risk of bias assessment

The quality of most of studies was poor or fair, with only 5 cohort studies, 4 case series and 1 cross-sectional study of good quality.

## 4. Discussion

The strengths of this meta-analysis are the size of the dataset, its broad range of sources, and the ability to examine movement disorder by their individual AE antibody associations. Movement disorders are common in AE, though they usually occur in conjunction with neuropsychiatric disturbances or epilepsy. Characterization of these clinical features has lacked focus for a number of reasons. AE itself has a low incidence, and large case series are relatively scarce. Publications on AE have not employed consistent semantics for movement abnormalities. Previous review writers comment on this difficulty (7, 59). Certain types of movement disorders can be enormously helpful and may be the key to early AE diagnosis (6, 7, 59). Yet this needs to be seen in the context of the many other causes of the same movement disorders that do not involve autoimmunity. This study provides comprehensive information about the frequency and type of movement disorders in AE.

### 4.1. Occurrence and classification of movement disorders in all seropositive AE

Approximately half of adult patients diagnosed with seropositive AE experienced a movement disorder during their disease course. Dyskinesia was the most frequent classification. The generic definition of dyskinesia that we employed could possibly have captured a wider range of movement disorders. Another factor was that NMDAR mediated AE, where a variety of hyperkinetic movements that can broadly be termed dyskinetic occur, made up half of all AE cases. Imprecise classification in some of the larger studies may have contributed to the failure of common movement disorders, including dystonia, myoclonus, tic, and stereotypy, to reach the threshold for inclusion in the meta-analysis.

#### 4.1.1. NMDAR mediated AE

Motor disturbances are often present in this type of AE. Our meta-analysis identified at least one movement disorder in 62% of patients, with dyskinesia being the most frequent. Previous studies have estimated the occurrence of movement disorders in NMDAR mediated AE as high as 90%, though this statistic is influenced by the inclusion of pediatric cases, where movement disorders are a common and early feature (7). In adults, this AE often presents with psychiatric symptoms, with movement disorders being somewhat less frequent (10). The mixed or transitional character of involuntary movement in this condition has proved difficult to classify, even for an expert panel of movement disorder specialists (7). This particularly applies to repetitive involuntary movements—grimacing or chewing orofacial activity; limb movements with cyclical, ballistic or complex patterns—where dyskinetic and stereotypic terms overlap (60). One previous study suggested that hypokinetic motor deficits—parkinsonian or catatonic—are a feature of adult NMDAR AE, (61) although we did not detect such a pattern. Overall, our findings agree with smaller, more detailed reports on motor phenomenology in NMDAR-positive cases, which emphasize mixed hyperkinetic disorders (7).

Based on animal studies, it has been postulated that NMDA receptor internalization occurs in this type of AE, leading to a state of NMDA receptor hypofunction (62, 63). This reduces GABA synthesis. The combination of NMDA receptor and GABA underactivity could lead to impaired inhibition of pyramidal neurons, especially in the hippocampal formation, which in turn increases dopamine release from nigral neurons and results in dyskinetic movements (63).

Psychiatric symptoms and seizures were the dominant non-motor features in this group, in agreement with a prior systematic review (64). That study identified that the presence of dyskinesia predicted the diagnosis of NMDAR mediated AE in 87% of cases, more than for psychiatric symptoms (64).

#### 4.1.2. LGI1 mediated AE

We confirmed that FBDS accounts for the majority of movement disorders in LGI1 AE, where it is a pathognomic clinical feature (65). Whether FBDS is actually a movement disorder, as opposed to a form of tonic seizure activity, is debated (66). The mechanism of FBDS is unknown, though neuroimaging studies show that it arises from the motor cortex and the basal ganglia (38, 67). One possible explanation involves altered neuronal excitability and synaptic transmission from targeting of the LGI1 component of the voltage-gated potassium channel complex (67). Cognitive deficits, at high prevalence, were the commonest associated clinical feature, followed by seizures. While another review paper found more psychiatric symptoms with LGI1 antibodies, (68) our conclusion was that this disorder has a weaker psychobehavioural flavor than NMDAR AE.

#### 4.1.3. CASPR2 mediated AE

Nearly half of these patients had one or more movement disorder, ataxia being the commonest. As with LGI1 mediated AE, cognitive effects and seizures were often present. These are similar findings to previous studies (26, 69). Ataxia in CASPR2-related antibody syndromes is believed to be caused by immune-mediated voltage-gated potassium ion channel dysfunction at the nodes of Ranvier (70).

#### 4.1.4. Anti GABA antibody mediated AE

Movement disorders in this subtype were far less common, whilst seizures and psychiatric disturbances were frequent. The low occurrence of movement disorders is somewhat surprising given GABA is the major inhibitory neurotransmitter in the basal ganglia and its interrupted function would be expected to result in a range of motor abnormalities (71). The low number of studies describing this rarer condition means the findings must be interpreted with caution. The need to combine GABA-A and GABA-B receptor antibody detection into one category because of low case numbers may have obscured serotype-specific variations.

#### 4.1.5. Other surface antibody mediated AE

The methodology of our paper did not capture rare and more recently identified subtypes of antibody-mediated AE that can cause movement disorders. One such condition is anti-IgLON5 disease, first reported in 2014, and unique in that it is considered an autoimmune neurodegenerative disease (72). Only two publications met the inclusion criteria for this meta-analysis. In one paper, 6 of the 13 patients (46.2%) had a movement disorder not otherwise specified (41). The other described cerebellar features (52; 72.2%) and choreoathetosis (24; 33.3%) (25). Bulbar symptoms, sleep dysfunction and a motor deficit resembling progressive supranuclear palsy are also frequently present in anti-IgLON5 disease (73).

#### 4.1.6. Seronegative AE

An important though problematic area in this field is the entity of seronegative AE, which may account for up to 50% of all AE (74). A 2016 consensus paper proposed a set of diagnostic guidelines that do not rely on antibody status, providing a basis for identification of seronegative cases (75). The criteria for possible AE are relatively permissive, while those for definite AE focus on limbic involvement with seizures and neuropsychiatric deficits. While we conceived this as a meta-analysis of antibody-positive AE, information on movement disorders in seronegative AE is available from a recent study of 147 cases diagnosed according to the 2016 criteria (76). Involuntary movements were present in 25% of patients and cerebellar deficits in 80%, suggesting that movement disorders occur at roughly comparable rates to seropositive AE. Subject to further research, there may be an argument for including new onset movement disorder as a secondary diagnostic criterion for possible seronegative AE.

### 4.2. Other clinical correlations of movement disorders in seropositive AE

We found that higher occurrence of movement disorders was associated with other clinical features, including psychiatric symptoms, and abnormalities in conscious state, sleep and speech. A high rate of movement disorder had a dichotomous prognostic implication—mortality was increased, though post treatment mRS scores were lower with less relapses. A possible explanation for these contradictory findings involves time of diagnosis of AE. The presence of movement disorders may allow earlier diagnosis and hence more rapid institution of therapy, which has been shown to improve outcomes (77). But if prominent movement disorders also denote more severe disease, patients with a delayed diagnosis would tend to fare worse.

In the absence of detailed individual patient data, these association findings can only indicate possible relationships. They appear to suggest that movement abnormalities may not only provide diagnostic clues but also be prognostic indicators. The value of movement disorders in AE diagnosis or prognosis has not previously been systematically evaluated.

### 4.3. Study limitations

As highlighted already, variable standards were applied to movement disorder reporting and definitions, which may have affected accuracy of some interpretations. All diagnoses of AE in eligible papers were accepted as correct, though some non-AE cases may have been included. The absence of seronegative AE cases restricts generalizability to all AE. Our findings were also limited by modest numbers of cases for the rarer AE subtypes. Most studies were retrospective and followed poor or fair methodology that possibly allowed recall bias. The absence of individual patient data also limits the extent to which the prognostic value of movement disorders in AE can be inferred. Some patients may have been on antipsychotic treatment and had drug-induced movement disorders, a factor that future researchers should consider when reporting motor abnormalities. Our methodology may have captured pediatric cases or excluded adults in mixed age group surveys that did not provide separate data.

## 5. Conclusions

The findings of our meta-analysis are of clinical significance, quantifying and classifying the occurrence of movement disorders in different cell surface antibody AE subtypes. Future clinical studies of AE should attempt to describe more clearly the typology of movement disorders, and their association with functional outcomes. It would also be advantageous to have better longitudinal reporting about movement disorders—at time of diagnosis, changes in patterns and severity across the disease course, and temporal relationships to immunotherapy.

## Data availability statement

The raw data supporting the conclusions of this article will be made available by the authors, without undue reservation.

## Author contributions

PS and LM performed the abstract and full text screening, as well as data extraction. MM resolved any conflicts. ZC performed statistical analysis. MM and PK provided supervision and guidance on manuscript preparation. All authors were involved in the design of the study and contributed to the writing of the manuscript. All authors contributed to the article and approved the submitted version.
