# Supplementary material for: Movement disorders in cell surface antibody mediated autoimmune encephalitis: a meta-analysis
Source: Front Neurol. 2023 Jul 21;14:1225523. doi: 10.3389/fneur.2023.1225523 (PMC10401600; doi:10.3389/fneur.2023.1225523)
Supplement: Supplementary file 1 [file Table_1.DOCX]

Supplementary Table 1: Quality of studies assessment

| Study quality | Case series | Cohort/case-control/cross-sectional |
| --- | --- | --- |
| Good | 14 or more points | 3-4 stars in selection domain AND 1-2 stars in comparability domain AND 2-3 stars in outcome/exposure domain. |
| Fair |  | 2 stars in selection domain AND 1-2 stars in comparability domain AND 2-3 stars in outcome/exposure domain. |
| Poor | Under 14 points | 1 star in selection domain OR 0 stars in comparability domain OR 0-1 star in outcome/exposure domain. |

Supplementary Table 2: Detailed demographic data

| **Study (first author; year)** | **Country** | **Study type** | **Age average years (SD)** | **Sex (F;M)** | **Study duration** | **Mean duration of follow up** | **Antibody** | **Paper quality** |
| --- | --- | --- | --- | --- | --- | --- | --- | --- |
| Celicanin et al 2017 | Denmark | Retrospective cohort | Mean 62 (29-84) | 9F;7M | N/A | Median 3.2 years | 16 LGI1 | Good |
| Dong et al 2019 | China | Retrospective cohort | Mean 50.7 | 18F; 8M | 2.5 years | 1 month |  | Fair |
|  |  |  | Mean 39.4 | 10F; 4M |  |  | 14 NMDAR |  |
|  |  |  | Mean age 53.7 | 4F; 3M |  |  | 7 LGI1 |  |
|  |  |  | Mean 64.6 | 4F; 1M |  |  | 5 GABA |  |
| Elkhider et al 2021 | US | Retrospective cohort | Mean 47 years (25-81) | 5F, 5M | 5.5 years | N/A |  | Fair |
|  |  |  | Mean 35 (25-44) | 4F, 1M |  |  | 5 NMDAR |  |
|  |  |  | Mean 72 (62-81) | 1F, 2M |  |  | 3 LGI1 |  |
|  |  |  | Mean 38 | 2M |  |  | 2 GAD |  |
| Etemadifar et al 2021 | Iran | Cross sectional | Mean 34.9 (12.8) | 21F, 18M | N/A | 34.7+/-13.5 months |  | Fair |
|  |  |  | Mean 33.2 (11.3) | 17F, 9M |  | 30.6+/-13.3 months | 26 NMDAR |  |
|  |  |  | Mean 32.75 | 2F, 6M |  | mean 46.5 months | 8 GABA |  |
|  |  |  | Mean 30 | 1F |  | 48 months | 1 GAD |  |
|  |  |  | Mean 52.25 | 1F, 3M |  | 34.5 mean months | 4 anti Zic4 |  |
| Gaig et al 2021 | Spain | Retrospective cohort | Median 62 (42-91) | 32F; 40M | 6.5 years | 3 months | 72 IGLON5 | Fair |
| Guery et al 2022 | France | Retrospective cohort | Mean 63 (13.6) | 14F; 25M | 14 years | Median 4 2 months | 39 LGI1 | Fair |
| Guo et al 2022 | China | Prospective cohort | Unknown | Unknown | 7 years |  |  | Good |
|  |  |  | Median 44 (range 18-82) | 25F, 19M |  | 44 months median | 44 LGI1 |  |
|  |  |  | Median 43 (range 14-64) | 19F, 16M |  | 18 months median | 35 CASPR |  |
|  |  |  | Median 44 (range 36-58) | 5F |  |  | 5 CASPR/LGI1 dual |  |
| Hayden et al 2021 | Hungary | Retrospective cohort | Mean 39.3 (1-75) | 11F; 19M | 6 years | 33 months |  | Poor |
|  |  |  | Mean 32.5 (1-75) | 8F; 11M |  |  | 19 NMDAR |  |
|  |  |  | Mean 46.8 (3-65) | 1F, 5M |  |  | 6 LGI1 |  |
|  |  |  | Mean 47 (16-67) | 1F, 2M |  |  | 3 CASPR |  |
|  |  |  | Mean 47.7 (3-72) | 1F, 2M |  |  | 3 GABA |  |
| Hirose et al 2021 | Japan | Retrospective cohort | Median 20.5 (range 3-59) | 49F; 7M | N/A | Median 45 months | 56 NMDAR | Poor |
| Hoftberger et al 2013 | US and Spain | Retrospective cohort | Median 61.5 (range 16-77) | 8F; 12M | 3.5 years | 11.8 mean months | 20 GABA | Good |
| Jiang et al 2021 | China | Retrospective cohort | Mean 24.33 (5.12) | Unknown | N/A | 5.5 years | 21 NMDAR | Good |
| Jia et al 2022 | China | Retrospective cohort | Unknown | 32F; 48M | N/A | N/A | 80 LGI1 | Poor |
| Joubert et al 2016 | France | Retrospective cohort | Median 64.5 (range 53-75) | 1F; 17M | 6 years | Median 43 months | 18 CASPR | Good |
| Kamble et al 2015 | India | Retrospective cohort | Unknown | Unknown | 3.5 years | N/A |  | Poor |
|  |  |  | Mean 21 | 4F |  |  | 4 NMDAR |  |
|  |  |  | Mean 57 | 1F; 1M |  |  | 2 VGKC |  |
|  |  |  | Mean 46.5 | 3F, 1M |  |  | 4 Anti TPO |  |
| Li et al 2022 | China | Retrospective cohort | Median 57 (IQR 45-66) | 30F; 61M | N/A | Median months 25 |  | Fair |
|  |  |  | Median 57 (IQR 43-65) | 24F; 52M |  | 29 months median | 76 LGI1 |  |
|  |  |  | Median 27 (IQR 14-43) | 6F; 9M |  | 20 months median | 15 CASPR |  |
| Lin N et al 2019 | China | Retrospective cohort | Median 21 (IQR 17-28) | 49F; 30M | 5 years and 10 months | N/A | 79 NMDAR | Poor |
| Liu X et al 2020 | China | Retrospective cohort | Median 52.7 (range 36-70) | 1F; 15M | 4 years | N/A | 16 LGI1 | Poor |
| Lizcano-Meneses et al 2021 | Brazil | Cross-sectional | Unknown |  | 6 years | N/A |  | Fair |
|  |  |  | Unknown | 12F; 6M |  |  | 18 NMDAR |  |
|  |  |  | Unknown |  |  |  | 2 LGI1 |  |
|  |  |  | Unknown |  |  |  | 2 GAD |  |
| Mizoguchi et al 2022 | Japan | Retrospective case control | Median 21 (range 16-50) | 8F, 1M | 6.5 years | 23 months median | 9 NMDAR | Fair |
|  |  |  | Median 38 (range 17-71) | 6F, 6M |  | 14.5 months median | 12 AE not otherwise specified |  |
| Muniz-Castrillo et al 2020 |  |  | Unknown |  | 8.5 years |  | 56 CASPR | Fair |
| Ni et al 2022 | China | Retrospective cohort | Median 60 (range 33-73) | 6F; 7M | 4 years and 5 months | Median 36 weeks | 13 IGLON5 | Good |
| Qiao et al 2021 | China | Retrospective cohort | Median 57 (IQR 52-67) | 36F; 81M | 4.75 years | Median 33 months | 117 LGI1 | Good |
| Schmitt et al 2012 | US | Retrospective cohort | Median 24 (range 18-55) | 19F, 4M | 6 years | 44 days | 23 NMDAR | Poor |
| Shan et al 2021 | China | Retrospective cohort | Median 35 (range 1-87) | 363F; 494M | 5 years | N/A |  | Good |
|  |  |  | Unknown | 252F; 242M |  |  | 494 NMDAR |  |
|  |  |  | Unknown | 54F; 106M |  |  | 160 LGI1 |  |
|  |  |  | Unknown | 31F; 52M |  |  | 83 GABA |  |
|  |  |  | Unknown | 18F; 8M |  |  | 26 CASPR |  |
| Shin et al 2020 | South Korea | Retrospective cohort | Unknown |  | 6 years and 9 months | N/A | 68 NMDAR | Poor |
| Tominaga et al 2018 | Japan | Retrospective cohort | Median 27 (range 12-47) | 28F; 6M | 18 years 9 months | N/A | 34 NMDAR | Poor |
| van Sonderen et al 2016 | Spain and Netherlands | Retrospective cohort | Median 66 (range 25-77) | 4F; 34M | 21 years | Median 36 months | 38 CASPR | Poor |
| Wagner et al 2018 | Austria | Retrospective cohort | Median 57 (range 13-73) | 8F; 9M | 10 years | Median 920 days | 17 NMDAR | Poor |
| Wang et al 2015 | China | Retrospective cohort | Median 21.6 (range 9-39) | 32F, 19M | 3 years | N/A | 51 NMDAR | Poor |
| Wickramasinghe et al 2021 | Sri Lanka | Prospective cohort | Mean 19.3 (11.9) | 22F, 7M | 12 months | N/A | 29 NMDAR | Poor |
| Wu et al 2020 | China | Prospective cohort | Mean 34.29 (14.86) | 36F;26M | 2 years | N/A | 62 NMDAR | Good |
| Yang et al 2020 | China | Retrospective cohort | Median 29 (14-65) | 42F; 36M | 7 years | N/A | 78 NMDAR | Fair |
| Yu et al 2020 | China | Retrospective cohort | Mean 30.04 (12.56) | 40F; 33M | 6 years | 2 months | 73 NMDAR | Fair |
| Zhang X et al 2019 | China | Retrospective cohort | Median 34 (range 13-54) | 18F;11M | 3.5 years | 1 year | 29 NMDAR | Poor |
| Zhang Y et al 2018 | China | Retrospective cohort | Mean 27.7 (SD 13.7) | 52F;59M | 5.5 years | 6-64 months | 111 NMDAR | Poor |
| Zhong R et al 2022 | China | Retrospective cohort | Median 52 (IQR 33-66) | 46F; 54M | 6 years and 8 months | Median 18 months |  | Poor |
|  |  |  | Unknown | 18F; 22M |  |  | 40 NMDAR |  |
|  |  |  | Unknown | 11F; 13M |  |  | 24 GABA |  |
|  |  |  | Unknown | 17F; 9M |  |  | 36 LGI1 |  |
| Gövert | Spain and Germany | Retrospective cohort | Median 68 (range 20-85) | 17F, 132M | 25.5 years | Median 47 days | 149 NMDAR | Fair |
|  |  |  | Median 58 (range 41-78) | 3F, 12M |  | Median 47 days | 15 LGI1/CASPR |  |
|  |  |  | Median 65 (range 22-86) | 46F, 59M |  | Median 6 days | 105 LGI1 |  |
| Sasikumar | India | Retrospective | Unknown | Unknown |  | N/A | 23 LGI1 | Poor |

| Supplementary Table 3. Effects of demographics and autoimmune encephalitis sub-types on the proportions and heterogeneities of the movement disorders | | | | | | | | |
| --- | --- | --- | --- | --- | --- | --- | --- | --- |
|  |  | **Any movement disorders (min)** | **Any movement disorders (max)** | **Ataxia** | **Chorea** | **FBDS** | **Involuntary movements** | **Parkinsonism** |
| **Age** | Coefficient | -0.01 | -0.01 | 0.02 | -0.02 | 0.02 | -0.04 | -0.01 |
|  | (95% CI) | (-0.02–0.00) | (-0.02–0.01) | (-0.02–0.05) | (-0.06–0.02) | (-0.00–0.04) | (-0.05–-0.02) | (-0.02–0.00) |
|  | *p*-value | 0.066 | 0.39 | 0.31 | 0.25 | 0.11 | <0.001 | 0.21 |
|  | *R^2^* | 0% | 0% | 0% | 1% | 0% | 70% | 38% |
| **Proportion of Females** | Coefficient | 0.01 | 0.01 | -0.00 | 0.05 | -0.01 | 0.03 | 0.01 |
|  | (95% CI) | (0.00–0.02) | (-0.00–0.02) | (-0.02–0.02) | (0.02–0.06) | (-0.02–0.00) | (0.01–0.05) | (-0.00–0.02) |
|  | *p*-value | 0.009 | 0.067 | 0.96 | <0.001 | 0.21 | 0.001 | 0.25 |
|  | *R^2^* | 12% | 0% | 0% | 90% | 0% | 53% | 34% |
| **Year of Publication** | Coefficient | -0.03 | -0.03 | 0.01 | -0.66 | -0.01 | -0.09 | 0.07 |
|  | (95% CI) | (-0.09–0.03) | (-0.12–0.06) | (-0.10–0.12) | (-1.71–0.40) | (-0.13–0.11) | (-0.25–0.07) | (-0.46–0.60) |
|  | *p*-value | 0.36 | 0.47 | 0.88 | 0.23 | 0.88 | 0.26 | 0.79 |
|  | *R^2^* | 5% | 0% | 0% | 0% | 0% | 8% | 0% |
| **AE Sub-types** | *p*-value | 0.084 | 0.52 | 0.56 | 0.42 | 0.004 | <0.001 | 0.69 |
|  | *R^2^* | 17% | 0% | 0% | 0% | 54% | 72% | 0% |
| anti-GABAR vs anti-CASPR2 | Coefficient | -0.69 | -0.69 | -0.69 | - | - | - | - |
|  | (95% CI) | (-1.91–0.53) | (-2.54–1.15) | (-2.00–0.62) |  |  |  |  |
|  | HB-corrected *p*-value | 1.00 | 1.00 | 0.90 |  |  |  |  |
| anti-LGI1 vs anti-CASPR2 | Coefficient | 0.01 | 0.24 | - | - | - | - | - |
|  | (95% CI) | (-0.79–0.81) | (-0.98–1.46) |  |  |  |  |  |
|  | HB-corrected *p*-value | 0.97 | 1.00 |  |  |  |  |  |
| Mixed vs anti-CASPR2 | Coefficient | 0.04 | 0.45 | -0.11 | - | - | - | - |
|  | (95% CI) | (-0.70–0.78) | (-0.68–1.57) | (-0.97–0.75) |  |  |  |  |
|  | HB-corrected *p*-value | 1.00 | 1.00 | 0.80 |  |  |  |  |
| anti-NMDAR vs anti-CASPR2 | Coefficient | 0.42 | 0.53 | - | - | - | - | - |
|  | (95% CI) | (-0.32–1.17) | (-0.61–1.66) |  |  |  |  |  |
|  | HB-corrected *p*-value | 1.00 | 1.00 |  |  |  |  |  |
| anti-LGI1 vs anti-GABAR | Coefficient | 0.70 | 0.93 | - | - | - | - | - |
|  | (95% CI) | (-0.37–1.78) | (-0.70–2.56) |  |  |  |  |  |
|  | HB-corrected *p*-value | 1.00 | 1.00 |  |  |  |  |  |
| Mixed vs anti-GABAR | Coefficient | 0.73 | 1.14 | 0.58 | - | - | - | - |
|  | (95% CI) | (-0.30–1.77) | (-0.42–2.70) | (-0.57–1.73) |  |  |  |  |
|  | HB-corrected *p*-value | 1.00 | 1.00 | 0.64 |  |  |  |  |
| anti-NMDAR vs anti-GABAR | Coefficient | 1.12 | 1.22 | - | - | - | - | - |
|  | (95% CI) | (0.08–2.15) | (-0.35–2.79) |  |  |  |  |  |
|  | HB-corrected *p*-value | 0.35 | 1.00 |  |  |  |  |  |
| Mixed vs anti-LGI1 | Coefficient | 0.03 | 0.21 | - | 0.53 | -0.37 | 0.41 | -0.29 |
|  | (95% CI) | (-0.44–0.50) | (-0.52–0.93) |  | (-0.76–1.82) | (-0.73–-0.01) | (-0.50–1.31) | (-0.99–0.41) |
|  | HB-corrected *p*-value | 0.90 | 1.00 |  | 0.42 | 0.092 | 0.38 | 1.00 |
| anti-NMDAR vs anti-LGI1 | Coefficient | 0.41 | 0.29 | - | - | -1.02 | 1.40 | -0.34 |
|  | (95% CI) | (-0.06–0.89) | (-0.45–1.02) |  |  | (-1.67–-0.38) | (0.56–2.24) | (-1.23–0.55) |
|  | HB-corrected *p*-value | 1.00 | 1.00 |  |  | 0.006 | 0.002 | 0.92 |
| anti-NMDAR vs Mixed | Coefficient | 0.38 | 0.08 | - | - | -0.66 | 0.99 | -0.04 |
|  | (95% CI) | (0.02–0.75) | (-0.48–0.65) |  |  | (-1.31–0.00) | (0.43–1.55) | (-0.72–0.63) |
|  | HB-corrected *p*-value | 0.72 | 0.774 |  |  | 0.050 | <0.001 | 0.90 |
| AE, autoimmune encephalitis; anti-CASPR2, anti-contactin associated protein-like 2; anti-GABAR, anti-gamma-aminobutyric-acid receptor; anti-LGI1, anti-leucine-rich glioma-inactivated 1; anti-NMDAR, anti-N-methyl-D-aspartate receptor; CI, confidence interval; FBDS, faciobrachial dystonic seizures | | | | | | | | |

Supplementary Table 4:

| **Associations between any movement disorder and outcome measures** | | | | | | |
| --- | --- | --- | --- | --- | --- | --- |
|  | | **Pre-treatment mRS≤2** | **Post-treatment mRS≤2** | **Relapses** | **Mortality** | |
|  |  |  |  |  | **Raw** | **Adjusted with age and sex** |
| **Any movement disorder (min)** | **Coefficient** | 1.79 | 1.53 | -0.78 | 1.23 | 1.61 |
|  | **(95% CI)** | (0.26–3.32) | (0.32–2.73) | (-1.22–-0.33) | (-0.36–2.83) | (0.50–2.71) |
|  | **p-value** | 0.022 | 0.013 | <0.001 | 0.13 | 0.004 |
| **Any movement disorder (max)** | **Coefficient** | -0.74 | 1.81 | -0.69 | 1.18 | 1.26 |
|  | **(95% CI)** | (-3.47–1.99) | (1.20–2.41) | (-1.06–-0.31) | (0.16–2.21) | (0.69–1.83) |
|  | **p-value** | 0.60 | <0.001 | <0.001 | 0.024 | <0.001 |
| CI, confidence interval | | | | | |  |

Supplementary Figure 1a: Funnel plot of papers reporting any movement disorders (minimum prevalence)

Supplementary Figure 1b: Funnel plot of papers reporting any movement disorders (maximum prevalence)
