# Supplementary material for: Movement disorders in cell surface antibody mediated autoimmune encephalitis: a meta-analysis
Source: Front Neurol. 2023 Jul 21;14:1225523. doi: 10.3389/fneur.2023.1225523 (PMC10401600; doi:10.3389/fneur.2023.1225523)
Supplement: Supplementary file 2 [file Data_Sheet_1.docx]

**Appendix 1:**

**Database:**
Ovid MEDLINE(R) <1946 to August 10 2022>

| **#** | **Query** | **Results from 10 Aug 2022** |
| --- | --- | --- |
| 1 | movement disorders/ or dyskinesias/ or chorea/ or dystonic disorders/ | 26,114 |
| 2 | ataxia/ or athetosis/ or catalepsy/ or chorea/ or dystonia/ or hyperkinesis/ or hypokinesia/ or myoclonus/ or tremor/ | 42,333 |
| 3 | [ballismus.mp](about:blank). | 73 |
| 4 | Muscle Rigidity/ | 2,261 |
| 5 | myokymia/ or myotonia/ or spasm/ | 8,693 |
| 6 | Myoclonus/ | 5,412 |
| 7 | [parkinsonism.mp](about:blank). | 17,758 |
| 8 | 1 or 2 or 3 or 4 or 5 or 6 or 7 | 86,463 |
| 9 | Limbic Encephalitis/ | 1,061 |
| 10 | immune-mediated [encephalitis.mp](about:blank). | 70 |
| 11 | brainstem [encephalitis.mp](about:blank). | 565 |
| 12 | IgLON5.mp. | 97 |
| 13 | anti GAD65.mp. | 149 |
| 14 | anti [vgkc.mp](about:blank). | 56 |
| 15 | anti LGI1.mp. | 135 |
| 16 | Anti-N-Methyl-D-Aspartate Receptor Encephalitis/ | 1,331 |
| 17 | anti CASPR2.mp. | 57 |
| 18 | anti GABA.mp. | 154 |
| 19 | anti AMPA.mp. | 28 |
| 20 | anti D2.mp. | 58 |
| 21 | Anti Dopamine-2.mp. | 3 |
| 22 | Anti Glycine.mp. | 36 |
| 23 | Anti DPPX.mp. | 17 |
| 24 | Anti GlyR.mp. | 6 |
| 25 | Anti mGluR1.mp. | 13 |
| 26 | Anti mGluR5.mp. | 10 |
| 27 | Anti [amphiphysin.mp](about:blank). | 55 |
| 28 | seronegative autoimmune [encephalitis.mp](about:blank). | 13 |
| 29 | antibody negative autoimmune [encephalitis.mp](about:blank). | 9 |
| 30 | 9 or 10 or 11 or 12 or 13 or 14 or 15 or 16 or 17 or 18 or 19 or 20 or 21 or 22 or 23 or 24 or 25 or 26 or 27 or 28 or 29 | 3,665 |
| 31 | 8 and 30 | 223 |
| 32 | limit 31 to humans | 219 |

movement disorders/ or dyskinesias/ or chorea/ or dystonic disorders/
ataxia/ or athetosis/ or catalepsy/ or chorea/ or dystonia/ or hyperkinesis/ or hypokinesia/ or myoclonus/ or tremor/
[ballismus.mp](about:blank).
Muscle Rigidity/
myokymia/ or myotonia/ or spasm/
Myoclonus/
[parkinsonism.mp](about:blank).
1 or 2 or 3 or 4 or 5 or 6 or 7
Limbic Encephalitis/
immune-mediated [encephalitis.mp](about:blank).
brainstem [encephalitis.mp](about:blank).
IgLON5.mp.
anti GAD65.mp.
anti [vgkc.mp](about:blank).
anti LGI1.mp.
Anti-N-Methyl-D-Aspartate Receptor Encephalitis/
anti CASPR2.mp.
anti GABA.mp.
anti AMPA.mp.
anti D2.mp.
Anti Dopamine-2.mp.
Anti Glycine.mp.
Anti DPPX.mp.
Anti GlyR.mp.
Anti mGluR1.mp.
Anti mGluR5.mp.
Anti [amphiphysin.mp](about:blank).
seronegative autoimmune [encephalitis.mp](about:blank).
antibody negative autoimmune [encephalitis.mp](about:blank).
9 or 10 or 11 or 12 or 13 or 14 or 15 or 16 or 17 or 18 or 19 or 20 or 21 or 22 or 23 or 24 or 25 or 26 or 27 or 28 or 29
8 and 30
limit 31 to humans

**Database:**
Embase <1974 to 2022 August 10>

| **#** | **Query** | **Results from 10 Aug 2022** |
| --- | --- | --- |
| 1 | movement disorders/ or dyskinesias/ or chorea/ or dystonic disorders/ | 56,005 |
| 2 | ataxia/ or athetosis/ or catalepsy/ or chorea/ or dystonia/ or hyperkinesis/ or hypokinesia/ or myoclonus/ or tremor/ | 133,926 |
| 3 | [ballismus.mp](about:blank). | 165 |
| 4 | Muscle Rigidity/ | 10,279 |
| 5 | myokymia/ or myotonia/ or spasm/ | 14,809 |
| 6 | Myoclonus/ | 14,935 |
| 7 | [parkinsonism.mp](about:blank). | 42,631 |
| 8 | 1 or 2 or 3 or 4 or 5 or 6 or 7 | 225,830 |
| 9 | Limbic Encephalitis/ | 197 |
| 10 | immune-mediated [encephalitis.mp](about:blank). | 148 |
| 11 | brainstem [encephalitis.mp](about:blank). | 989 |
| 12 | IgLON5.mp. | 206 |
| 13 | anti GAD65.mp. | 344 |
| 14 | anti [vgkc.mp](about:blank). | 200 |
| 15 | anti LGI1.mp. | 323 |
| 16 | Anti-N-Methyl-D-Aspartate Receptor Encephalitis/ | 2,112 |
| 17 | anti CASPR2.mp. | 144 |
| 18 | anti GABA.mp. | 209 |
| 19 | anti AMPA.mp. | 62 |
| 20 | anti D2.mp. | 99 |
| 21 | Anti Dopamine-2.mp. | 3 |
| 22 | Anti Glycine.mp. | 75 |
| 23 | Anti DPPX.mp. | 25 |
| 24 | Anti GlyR.mp. | 24 |
| 25 | Anti mGluR1.mp. | 24 |
| 26 | Anti mGluR5.mp. | 23 |
| 27 | Anti [amphiphysin.mp](about:blank). | 136 |
| 28 | seronegative autoimmune [encephalitis.mp](about:blank). | 45 |
| 29 | antibody negative autoimmune [encephalitis.mp](about:blank). | 18 |
| 30 | 9 or 10 or 11 or 12 or 13 or 14 or 15 or 16 or 17 or 18 or 19 or 20 or 21 or 22 or 23 or 24 or 25 or 26 or 27 or 28 or 29 | 5,034 |
| 31 | 8 and 30 | 1,130 |
| 32 | limit 31 to humans | 1,097 |

movement disorders/ or dyskinesias/ or chorea/ or dystonic disorders/
ataxia/ or athetosis/ or catalepsy/ or chorea/ or dystonia/ or hyperkinesis/ or hypokinesia/ or myoclonus/ or tremor/
[ballismus.mp](about:blank).
Muscle Rigidity/
myokymia/ or myotonia/ or spasm/
Myoclonus/
[parkinsonism.mp](about:blank).
1 or 2 or 3 or 4 or 5 or 6 or 7
Limbic Encephalitis/
immune-mediated [encephalitis.mp](about:blank).
brainstem [encephalitis.mp](about:blank).
IgLON5.mp.
anti GAD65.mp.
anti [vgkc.mp](about:blank).
anti LGI1.mp.
Anti-N-Methyl-D-Aspartate Receptor Encephalitis/
anti CASPR2.mp.
anti GABA.mp.
anti AMPA.mp.
anti D2.mp.
Anti Dopamine-2.mp.
Anti Glycine.mp.
Anti DPPX.mp.
Anti GlyR.mp.
Anti mGluR1.mp.
Anti mGluR5.mp.
Anti [amphiphysin.mp](about:blank).
seronegative autoimmune [encephalitis.mp](about:blank).
antibody negative autoimmune [encephalitis.mp](about:blank).
9 or 10 or 11 or 12 or 13 or 14 or 15 or 16 or 17 or 18 or 19 or 20 or 21 or 22 or 23 or 24 or 25 or 26 or 27 or 28 or 29
8 and 30
limit 31 to humans

**Database:**
APA PsycInfo <1987 to August Week 1 2022>

| **#** | **Query** | **Results from 10 Aug 2022** |
| --- | --- | --- |
| 1 | movement disorders/ or dyskinesias/ or chorea/ or dystonic disorders/ | 3,910 |
| 2 | ataxia/ or athetosis/ or catalepsy/ or chorea/ or dystonia/ or hyperkinesis/ or hypokinesia/ or myoclonus/ or tremor/ | 14,705 |
| 3 | [ballismus.mp](about:blank). | 19 |
| 4 | Muscle Rigidity/ | 0 |
| 5 | myokymia/ or myotonia/ or spasm/ | 923 |
| 6 | Myoclonus/ | 953 |
| 7 | [parkinsonism.mp](about:blank). | 6,543 |
| 8 | 1 or 2 or 3 or 4 or 5 or 6 or 7 | 24,067 |
| 9 | Limbic Encephalitis/ | 0 |
| 10 | immune-mediated [encephalitis.mp](about:blank). | 14 |
| 11 | brainstem [encephalitis.mp](about:blank). | 82 |
| 12 | IgLON5.mp. | 30 |
| 13 | anti GAD65.mp. | 13 |
| 14 | anti [vgkc.mp](about:blank). | 10 |
| 15 | anti LGI1.mp. | 43 |
| 16 | Anti-N-Methyl-D-Aspartate Receptor Encephalitis/ | 0 |
| 17 | anti CASPR2.mp. | 20 |
| 18 | anti GABA.mp. | 13 |
| 19 | anti AMPA.mp. | 13 |
| 20 | anti D2.mp. | 9 |
| 21 | Anti Dopamine-2.mp. | 0 |
| 22 | Anti Glycine.mp. | 6 |
| 23 | Anti DPPX.mp. | 5 |
| 24 | Anti GlyR.mp. | 3 |
| 25 | Anti mGluR1.mp. | 5 |
| 26 | Anti mGluR5.mp. | 2 |
| 27 | Anti [amphiphysin.mp](about:blank). | 14 |
| 28 | seronegative autoimmune [encephalitis.mp](about:blank). | 5 |
| 29 | antibody negative autoimmune [encephalitis.mp](about:blank). | 1 |
| 30 | 9 or 10 or 11 or 12 or 13 or 14 or 15 or 16 or 17 or 18 or 19 or 20 or 21 or 22 or 23 or 24 or 25 or 26 or 27 or 28 or 29 | 267 |
| 31 | 8 and 30 | 28 |
| 32 | limit 31 to humans [Limit not valid in APA PsycInfo; records were retained] | 28 |

movement disorders/ or dyskinesias/ or chorea/ or dystonic disorders/
ataxia/ or athetosis/ or catalepsy/ or chorea/ or dystonia/ or hyperkinesis/ or hypokinesia/ or myoclonus/ or tremor/
[ballismus.mp](about:blank).
Muscle Rigidity/
myokymia/ or myotonia/ or spasm/
Myoclonus/
[parkinsonism.mp](about:blank).
1 or 2 or 3 or 4 or 5 or 6 or 7
Limbic Encephalitis/
immune-mediated [encephalitis.mp](about:blank).
brainstem [encephalitis.mp](about:blank).
IgLON5.mp.
anti GAD65.mp.
anti [vgkc.mp](about:blank).
anti LGI1.mp.
Anti-N-Methyl-D-Aspartate Receptor Encephalitis/
anti CASPR2.mp.
anti GABA.mp.
anti AMPA.mp.
anti D2.mp.
Anti Dopamine-2.mp.
Anti Glycine.mp.
Anti DPPX.mp.
Anti GlyR.mp.
Anti mGluR1.mp.
Anti mGluR5.mp.
Anti [amphiphysin.mp](about:blank).
seronegative autoimmune [encephalitis.mp](about:blank).
antibody negative autoimmune [encephalitis.mp](about:blank).
9 or 10 or 11 or 12 or 13 or 14 or 15 or 16 or 17 or 18 or 19 or 20 or 21 or 22 or 23 or 24 or 25 or 26 or 27 or 28 or 29
8 and 30
limit 31 to humans
